# 3’UTR-Seq analysis of chicken abdominal adipose tissue reveals widespread intron retention in 3’UTR and provides insight into molecular basis of feed efficiency

**DOI:** 10.1371/journal.pone.0269534

**Published:** 2022-07-01

**Authors:** Ziqing Wang, Mustafa Özçam, Behnam Abasht

**Affiliations:** Department of Animal & Food Sciences, University of Delaware, Newark, Delaware, United States of America; Sejong University, REPUBLIC OF KOREA

## Abstract

Feed efficiency (FE) is an important trait in the broiler industry due to its direct correlation to efficient muscle growth instead of fat deposition. The present study characterized and compared gene expression profiles in abdominal fat from broiler chickens of different FE levels to enhance the understanding of FE biology. Specifically, traditional whole-transcript RNA-sequencing (RNA-seq) and 3’ UTR-sequencing (3’ UTR-seq) were applied to 22 and 61 samples, respectively. Overall, these two sequencing techniques shared a high correlation (0.76) between normalized counts, although 3’ UTR-seq showed a higher variance in sequencing and mapping performance statistics across samples and a lower rate of uniquely mapped reads. A higher percentage of 3’ UTR-seq reads mapped to introns suggested the frequent presence of cleavage sites in introns, thus warranting future research to study its regulatory function. Differential expression analysis identified 1198 differentially expressed genes (DEGs) between high FE (HFE) and intermediate FE (IFE) chickens with False Discovery Rate < 0.05 and fold change > 1.2. The processes that were significantly enriched by the DEGs included extracellular matrix remodeling and mechanisms impacting gene expression at the transcriptional and translational levels. Gene ontology enrichment analysis suggested that the divergence in fat deposition and FE in broiler chickens could be associated with peroxisome and lipid metabolism possibly regulated by G0/G1 switch gene 2 (G0S2).

## Introduction

Modern commercial broiler chickens are genetically selected to be fast growing and have high muscle yield to meet increasing global demand for poultry meat [1]. In broiler chickens, feed efficiency (FE) is described as the ability to convert feed into body weight gain, and thus is an essential trait to the broiler industry. FE is affected by environmental, genetic, and nutritional factors, for example, dietary supplementation of a plant-extract antibiotic substitute was found to significantly improve breast muscle yield [2], which is a trait correlated with FE. FE also correlates with fatness in chickens. Not only does excessive fat accumulation diminish economic profits due to the decrease in FE and carcass yield, chickens of higher abdominal fat content also exhibit paler breast muscle with higher drip loss during cooking, rendering a harder meat texture [3]. Accordingly, improving FE and reducing adiposity in commercial broilers could generate economic benefits and improve sustainability of the broiler industry.

Furthermore, the chicken has been used extensively as a biomedical model to study adiposity, as the chicken shares key metabolic characteristics with humans. One commonality is that lipids in both chickens and humans are synthesized in the liver and then transported to the adipose tissue for storage and release [4,5]. This hepatic lipogenesis, in both chickens and mammals, is subjected to analogous hormonal and nutritional controls [6,7]. Akin to obese and type II diabetic patients, chickens are naturally hyperglycemic and possess innate insensitivity to insulin [6]. Therefore, a deeper understanding of adipose biology in chickens may also help to advance our knowledge of obesity and insulin resistance.

Global gene expression of chicken adipose tissue has been previously studied using microarray or RNA-seq technologies. The visceral fat of high-body-weight chickens showed higher expression of lipogenic genes and recruited more transcription factors to stimulate biosynthesis of fatty acids (FA) [8]. Resnyk et al. identified numerous up-regulated hemostatic and lipolytic genes as well as enhanced expression of gluconeogenesis or glycolysis genes in genetically lean chickens. In fat chickens, lipogenic, angiogenic and adipogenic genes were overexpressed [9]. Similarly, Zhuo et al. [10] revealed up-regulation of lipid synthesis and adipogenesis genes and down-regulation of genes related to lipid hydrolysis and adipose derived hormone synthesis in chickens with low FE.

3’ UTR sequencing (3’ UTR-seq) is a powerful and simple method to measure mRNA quantitatively through sequencing a small fragment (e.g., 100 bases) at the 3’ end of polyadenylated RNAs. This method has been proposed as a lower-cost alternative to RNA-seq to profile gene expression for differential expression (DE) analysis [11], as the number of reads generated by 3’ UTR-seq is expected to be proportional to the sample’s transcripts. Additionally, 3’ UTR-seq can bypass the biased estimation of expression levels in RNA-seq resulting from over-representation of long transcripts and save more sequencing space to increase the degree of multiplexing [11]. Various studies have established a comparable performance between 3’ UTR-seq and RNA-seq in DE analysis. In a study on human cardiomyocytes, Xiong et al. reported strong correlations of read counts and fold changes at the level of individual genes, as well as consistent results in biological interpretations, overlap in ranking of differentially expressed genes (DEGs) and gene signatures between the two techniques [12]. Moreover, for a non-model species that requires de novo transcriptome assembly, 3’ UTR-seq revealed more DE contigs than RNA-seq [13].

The purpose of this paper is to characterize the gene expression profile in broiler abdominal fat using 3’ UTR-seq to gain insights into biological and molecular pathways involved in adiposity as an associated trait with FE. DE and enrichment analysis showed that the divergence in fat deposition of broilers of distinct FE levels were associated with DEGs relevant to extracellular matrix (ECM) remodeling, peroxisome, fatty acid (FA) oxidation and triacylglycerol synthesis. This study also provides a comparison between RNA-seq and 3’ UTR-seq in a real-case scenario. To the best of our knowledge, there’s no published work using 3’ UTR-seq to profile gene expression in chicken adipose tissue at this time.

## Method and materials

### Experiment and tissue collection

The experiment involved 2400 randomly sampled commercial broiler chickens from 6 local farms in Delmarva region each from a different hatch of the same broiler cross [10]. In each hatch, 400 29-day-old chickens were sampled from one farm and transferred to an experimental station, where they were individually fed ad libitum as described previously [10].

At the beginning of the experiment (29 days of age), chicken body weight and individual feeder weight were measured. Sick and dead chickens were removed during the experiment. At 46 days of age, body weight and feeder weight measurements were collected a second time, and, at 47 days of age, the chickens were euthanized via manual cervical dislocation. Immediately following euthanasia, tissue sampling and weighing of body composition traits was performed. The ultimate pH (pHu) of pectoralis major muscle and the mass of abdominal fat were measured after keeping the carcasses at 4°C for 24 hours. About 1 gram of abdominal fat, defined as fat dissected from the abdominal cavity and around gizzard, was sampled and immediately frozen in liquid nitrogen and preserved at -80°C until RNA isolation could be performed. The experimental condition was in accordance with optimal industry growing standards and the protocols were approved by the University of Delaware Agricultural Animal Care and Use Committee.

### Estimation of feed efficiency

FE was measured by estimating residual feed consumption RFC, defined as the difference between actual and expected feed consumption, and was calculated using same formula implemented by Zhuo et al. 2015 [10]. After the first calculation, chickens with RFC outside of the mean ± 3 SDs were considered as outliers and excluded, after which the RFC was recalculated in the same way for the rest of the birds. Then the chickens were ranked by RFC within each hatch. For 3’UTR-seq, 61 chickens were selected from 6 hatches from the ends and middle of the distribution for RFC values and classified as low FE (LFE), high FE (HFE) and intermediate FE (IFE), respectively (S1 Table).

### Sample preparation and sequencing

Total RNA was extracted from approximately 70 mg of abdominal fat tissue using mirVana™ PARIS™ Kit (Life Technologies) through chemical extraction [14]. RNA concentration and integrity for each sample were checked by NanoDrop 1000 (Thermo Scientific) and Agilent Bioanalyzer 2100 (Agilent Technologies), respectively. cDNA libraries were constructed using QuantSeq 3’ mRNA-Seq Library Prep Kit for Illumina (reverse) sequencing, also known as the 3’ T-fill method [15,16]. First, the poly-A tails of the mRNAs from about 500 ng of total RNA were bound by primers containing poly-T oligonucleotides as well as Illumina-compatible sequences to initiate reverse transcription of the first strand cDNA. After degrading the RNA template, second-strand synthesis was initiated by random primers with Illumina-linker sequences at the 5’ end. The next step involved purification of the generated double-stranded cDNAs from reaction components through magnetic beads. Finally, during PCR amplification, the libraries were ligated to the complete adapter sequences necessary for cluster generation. The concentration and quality of the cDNA libraries were validated using NanoDrop 1000 and Agilent Bioanalyzer 2100. The cDNA libraries from 61 samples were normalized to the same concentration and pooled. The pooled sample was loaded into the cluster station (cBot, Illumina), where instead of the primer mix, the T-fill solutions (provided in the T-fill Add-on Kit for QuantSeq 3’ mRNA- Seq Libraries) were used for filling the poly-A stretch with unlabeled dTTPs. One lane of a flow cell was used for single-end sequencing for 100 cycles on the Illumina HiSeq 2000 System (Illumina, Inc., San Diego, CA) at the Delaware Biotechnology Institute, University of Delaware (Newark, DE).

### RNA-seq samples

Raw RNA-seq data was obtained from a previous study by Zhuo et al., 2015, available at NCBI Sequence Read Archive (Accession SRP058295). [10]. It consisted of adipose tissue samples from abdominal fat of 22 broiler chickens, 21 of which were in overlap with 3’ UTR-seq samples. They were sequenced using the Illumina Hiseq 2000 system on four lanes of a flow cell with a paired-end 2 x 75-cycle sequencing protocol [10]. One sample (39663) was deemed outlier in that study by hierarchical clustering and correlation analysis [10], it was thus omitted from the analysis. The resulting 20 RNA-seq samples were then compared against the corresponding 3’ UTR-seq samples on data quality and mapping performance as well as gene structure coverage by reads.

### Quality check and reads alignment

For downstream analysis, first raw sequence reads of both RNA-seq and 3’ UTR-seq underwent quality check using FastQC v0.11.9 [17]. And MultiQC v1.11 was used to analyze FastQC results [18]. Then they were mapped to the chicken reference genome Gallus_gallus-6a (Ensembl, database version 99). Hisat2 v2.2.0 [19], a splice-aware aligner, was used for mapping RNA-Seq reads to improve mapping accuracy in case of reads spanning across two exons. Specifically, it was set to report only concordant mappings for both successfully mapped paired-reads and used the “RNA-strandness RF” parameter to indicate the orientation of paired-end reads, with upstream reads being from the reverse strand. Conversely, a non-splice-aware aligner Bowtie2 v2.3.5.1 [20] was used for mapping 3’ UTR-seq reads, because these 100-nucleotide-reads were primarily generated from the very end of 3’UTR, and hence, they were not expected to span across exons. To improve efficiency and accuracy of read alignment, Bowtie2 was used in “very sensitive local alignment” mode and by suppressing the unaligned records using the “no-unal” option. Next, HTSeq v0.11.2 [21] was utilized to further categorize the mapped reads using default parameters except that for 3’ UTR-seq, count data was obtained both using feature type “exon” (default) and “gene”. Additionally, RSeQC v3.0.1 [22] was used with the default settings to examine read distribution and gene structure coverage differences between RNA-seq and 3’ UTR-seq. All these steps mentioned above were performed in Biomix server [23] provided by Delaware Biotechnology Institute, University of Delaware.

### Statistical analysis

Normalized counts of 3’UTR-seq and RNA-seq samples were obtained using DESeq2 by the median of ratios method accounting for sequencing depth and biological variability in transcriptome composition [24] and Pearson correlation was calculated using the R stats package (v3.5.2). For 3’ UTR-seq samples, phenotypic traits of different FE groups were compared performing Tukey’s HSD test [25] using R stats package (v3.5.2) with default parameters. For DE analysis, the samples were filtered to exclude low count genes based on group size and then normalized using edgeR v3.24.3 [26], followed by linear modeling and empirical Bayes moderation for DE analysis using Limma v3.38.3 [27]. Considering the 6 hatches in this experiment, the edgeR-Limma pipeline was applied as it allowed modeling of the hatch as a random effect via estimating the mean-variance relationship as a function of average log-counts and generating precision weights for each observation [28]. We also applied edgeR and DESeq2 for DE analysis with hatch as a fixed effect to adjust for the hatch effect, similar to a randomized block design. All DE analyses were conducted on the count data obtained using “exon” or “gene” feature types in HTSeq. A Venn Diagram was drawn also using the package Limma. All the correlation and statistical analyses were performed in R v3.5.2 [29]. Functional analysis of DEGs was conducted using ToppFun of ToppGene Suite, with a false discovery rate threshold of 0.05 [30].

## Results

### Comparison of 3’ UTR-seq and RNA-seq

FastQC results of both techniques showed very good quality, with the Phred score higher than 28. On average, the number of reads per sample of 3’ UTR-seq was 12.8 times lower than that of RNA-seq (Table 1). The average percentage of duplicated reads was higher for 3’ UTR-seq reads, while the average GC content was lower. As for mapping performance, the average unique alignment rate was 10% higher for RNA-seq samples, with 3’ UTR-seq samples having more multi-mapping reads, which were discarded by HTseq when obtaining feature counts. The difference in duplicated reads, GC content and multi-mapping rate could be explained by the presence of consensus sequences [31] and the U-rich upstream sequence elements of the polyadenylation signals [32]. Moreover, 3’UTR-seq had a higher variance for both sample and mapping statistics, as shown by their higher standard deviations in Table 1. Contrarily, RNA-seq delivered a more stable and even performance across the 20 samples.

**Table 1:** Comparison of Sample and Mapping statistics of 3’UTR-seq and RNA-seq.

	Sample Statistics						Mapping Statistics (%)					
	M seqs		% Dups		% GC		>1 time		Exactly 1 time		0 time	
Technique	*M*	*SD*	*M*	*SD*	*M*	*SD*	*M*	*SD*	*M*	*SD*	*M*	*SD*
3’UTR-Seq	2.5	1.2	61.9	0.07	40.1	0.02	17.7	4.4	77.0	5.1	5.3	1.2
RNA-Seq	32.0	1.0	31.6	0.03	49.0	0.01	1.5	0.8	87.3	1.3	11.2	0.9

M seqs: Number of million sequence reads; % Dups: Percentage of duplicated reads; % GC: Percentage of GC content; SD: Standard deviation. Means (M) of each statistic between the two techniques differ significantly (*p* < .05).

**Fig 1:**
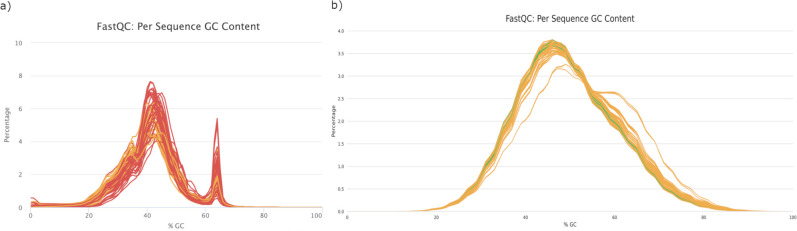
Percentage of reads against GC content. a) 3’ UTR-seq; b) RNA-seq.

Another peculiar difference pertinent to the GC content was a second unusual peak in 3’ UTR-seq samples (Fig 1). To determine which genes these high GC percentage reads belonged to, reads with a GC content between 62% and 66%, representing the edges around the second peak, were extracted using BBMap [33] and a subset of randomly selected 350 reads from these high GC reads were extracted for BLAST by FastqBLAST [34]. As a result, 98% of the extracted reads were mapped to 18S ribosomal RNA genes, suggesting that they were in fact rRNA instead of mRNA. Notwithstanding, these reads accounted for, on average, only 3% of the total reads—only a small portion of the total information. Similarly, it was found in humans that the 18S and 28S rRNAs have non-abundant polyadenylated transcripts, possibly resulting from degradation [35]. Therefore, this implies that the chicken 18S rRNA may also undergo a degradation process that adds short poly(A) tails to degradation intermediates, similar to humans and prokaryotes.

**Fig 2:**
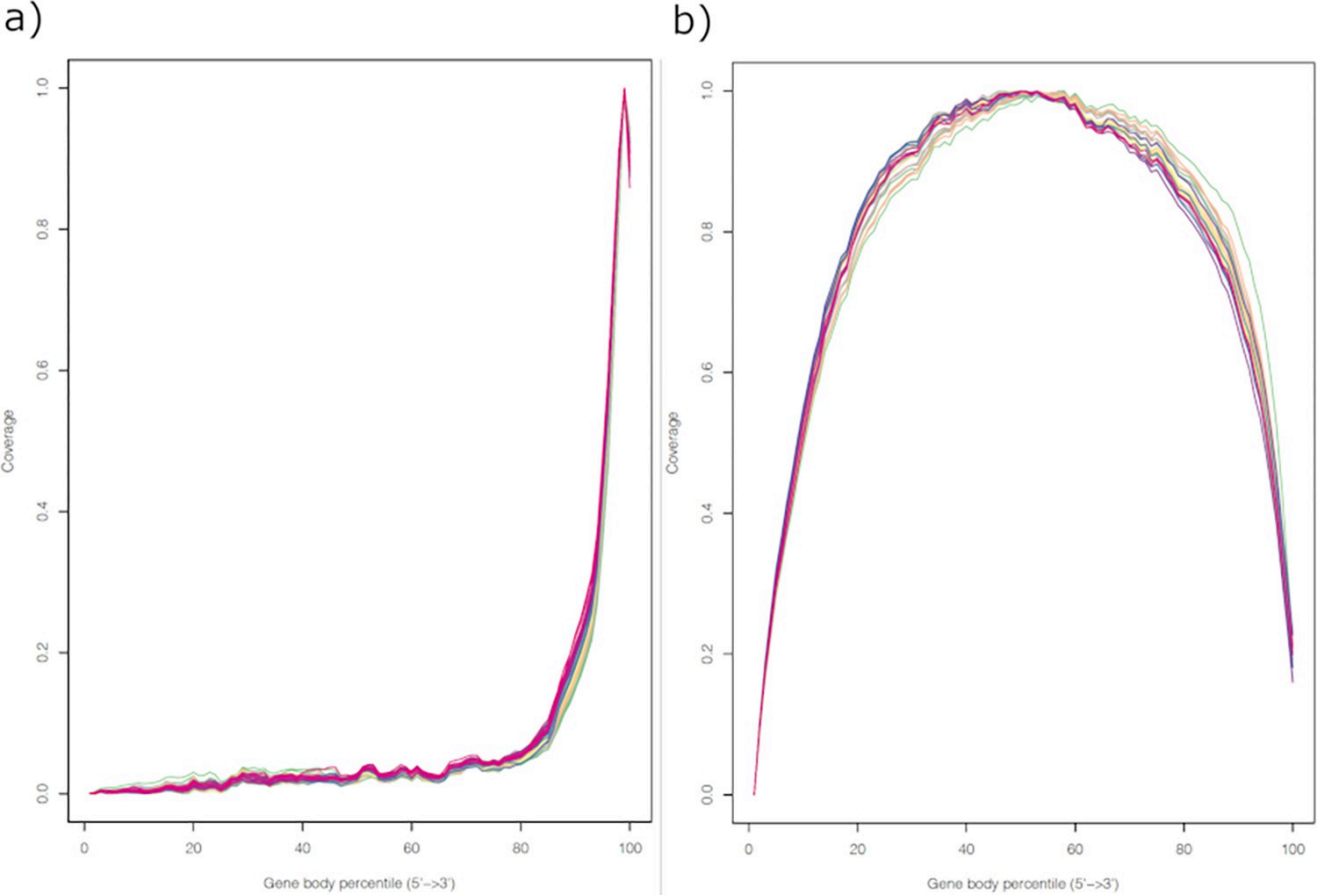
Gene body coverage. a) 3’ UTR-seq; b) RNA-seq.

Gene structure coverage of RNA-seq reads displayed a uniform pattern with low coverage at both 5’ and 3’ untranslated regions, while 3’ UTR-seq reads possessed high coverage at the 3’ end, as expected (Fig 2). As can be seen in Fig 2, both RNA-seq and 3’ UTR-seq techniques exhibited high consistency for gene structure coverage across all samples. Some 3’ UTR-seq reads were mapped to inner regions of the gene body, which may be attributed to limitations of the reference genome, alternative splicing and polyadenylation occurred in internal exons or introns [36].

**Fig 3:**
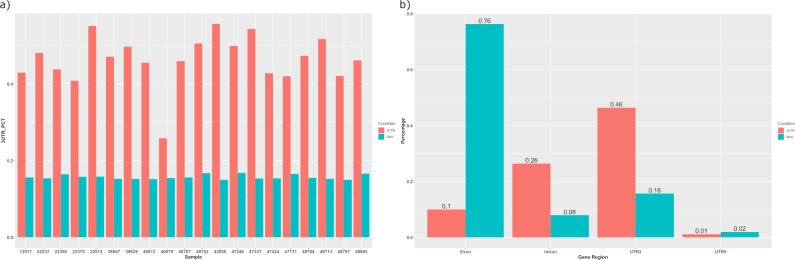
Read distribution results of RSeQC. a) 3’ UTR percentage (3UTR_PCT) of each sample; b) read distribution of gene structure shown in average percentage.

Read distribution results confirmed that on average 46% of 3’ UTR-seq reads are actually mapped to 3’ UTR, except one sample (40679) with only 25% (Fig 3A). The percentage reads mapped to 3’ UTR was on average 16% and 46% for RNA-seq and 3’ UTR-seq reads respectively, with a smaller variance among RNA-seq samples. Moreover, RNA-seq notably had most of its reads, with an average close to 80%, mapped to exons (Fig 3B). Surprisingly, 3’ UTR-seq had on average 26% of reads mapped to introns, 18% higher than RNA-seq, which reflected intron usage and its regulatory function as the 3’ UTR. Average Pearson correlation of normalized count between 3’ UTR-seq and RNA-seq samples was 0.76 (*p* < .001).

### Samples with possible muscle contamination

Hierarchical clustering of the 61 3’ UTR-seq samples confirmed that the inconsistency of the one sample (39663), separated from others on an isolated branch, was not due to technical problems but rather to the sampled tissue. Having excluded it, the remaining 60 samples were divided into three groups based on a histogram of RFC values. Specifically, birds with RFC smaller than -0.15 and larger than 0.19 were classified as HFE and LFE, respectively, and the ones in between as IFE.

DE analysis revealed some muscle related top DEGs when the HFE group was compared with the IFE group. For example, Myosin heavy chain 1E (MYH1E), actin alpha 1 (ACTA1), and Troponin T Type 3 (TNNT3) are genes related to contractile function and mainly expressed in skeletal muscle. Although not shown as outliers by clustering, examination of normalized counts disclosed 5 samples with much higher expression of these muscle related genes than others (S2 Table). Therefore, these 5 samples with possible muscle contamination were removed from this study for the sake of accuracy.

### Phenotypic traits

Correlation analysis between important production traits was conducted using all 2400 birds from the experiment (S1 Fig). Production traits like breast muscle and abdominal fat percentage were calculated as their weight divided by body weight. Weight at 46 days, the final weight at the end of the FE experiment (BW46), was highly correlated with feed consumption (FC) (r = 0.78) and weight gain (WG) (r = 0.81), while moderately correlated with breast muscle percentage (BMW%) (0.27). However, there was no correlation between final weight and abdominal fat percentage (FAT%). RFC had a moderate correlation with FAT% (r = 0.4) and BMW% (r = -0.3), indicating that chickens with lower FE are inclined to yield more abdominal fat and less breast muscle. This negative relationship between abdominal fat and breast muscle growth was further confirmed by their correlation coefficient (r = -0.26). Since RFC was accounted for initial and final body weight, its correlation with WG and bird final weight was zero. Feed conversion ratio (FCR), calculated as FC divided by weight gain, had a similar correlation with FAT% (r = 0.3) and BMW% (r = -0.31) as RFC. However, RFC may be a better indicator for FE in terms of abdominal fat content.

Between the sampled 55 birds, the difference in RFC across the three groups was found significant by Tukey’s HSD test (Table 2). In addition, other phenotypic traits including BMW%, FAT%, WG and bird final weight were also compared across the three FE groups. LFE chickens were found to have significant differences from IFE chickens in all traits, and from HFE chickens in all but the final body weight. Interestingly, FAT% is the only phenotype where the difference is significant across the three groups besides RFC, further indicating its association with FE. IFE and HFE birds were not found different in any other phenotypic traits except for RFC and FAT%, suggesting a comparable performance for the IFE group in production output, and probable disparity in feed cost.

**Table 2:** Results of Tukey HSD test for phenotypic traits between feed efficiency (FE) groups.

Variable	Group		p	95% confidence interval for mean	
				Lower bound	Upper bound
RFC	IFE	HFE	***	0.18	0.29
	LFE	HFE	***	0.54	0.65
	LFE	IFE	***	0.31	0.41
BMW%	IFE	HFE	-	-0.01	0.02
	LFE	HFE	**	-0.04	-0.007
	LFE	IFE	***	-0.04	-0.01
FAT%	IFE	HFE	*	0.009	0.68
	LFE	HFE	***	0.45	1.1
	LFE	IFE	**	0.13	0.78
WG (kg)	IFE	HFE	-	-0.1	0.16
	LFE	HFE	*	-0.28	-0.01
	LFE	IFE	**	-0.3	-0.05
Final weight (kg)	IFE	HFE	-	-0.06	0.29
	LFE	HFE	-	-0.29	0.07
	LFE	IFE	**	-0.39	-0.05

LFE: Low FE; HFE: High FE; IFE: Intermediate FE. Significance level: p-value < .05 *, < .01 **, < .001 ***, > .05.

### Identification and functional analysis of DEGs

Given that the IFE group was composed of chickens only from 2 out of 6 hatches (S1 Table), comparisons involving IFE were restricted to chickens from the two hatches, while comparison between HFE and LFE used chickens from all 6 hatches. Limma identified no DEGs comparing LFE vs. IFE as well as HFE vs. LFE groups, and 1198 DEGs between HFE and IFE, with a fold change greater than 1.2 and false discovery rate threshold smaller than 0.05. Among the DEGs, 709 were up-regulated and 489 were down-regulated in HFE birds compared to IFE ones. Table 3 listed the top 10 up- and down-regulated genes in the HFE group.

**Table 3:** Top 10 up- and down-regulated genes in HFE group.

Ensembl ID	Gene Symbol	Gene Full Name	Log2FC
**UP-regulated genes**			
ENSGALG00000014467	COPS7A	COP9 signalosome subunit 7A	↑2.6
ENSGALG00000023407	ZBTB34	Zinc finger and BTB domain containing 34	↑2.1
ENSGALG00000029540	MMRN2	Multimerin 2	↑2.0
ENSGALG00000032618	DUSP5	Dual specificity phosphatase 5	↑1.9
ENSGALG00000038599	PMM1	Phosphomannomutase 1	↑1.9
ENSGALG00000002486	RNF123	Ring finger protein 123	↑1.7
ENSGALG00000014616	MT3	Metallothionein 3	↑1.5
ENSGALG00000037995	STRIP1	Striatin interacting protein 1	↑1.5
ENSGALG00000002138	HYAL2	Hyaluronoglucosaminidase 2	↑1.5
ENSGALG00000038721	MAP1LC3A	Microtubule-associated protein 1 light chain 3 alpha	↑1.5
**Down-regulated genes**			
ENSGALG00000027707	ESM1	Endothelial cell specific molecule 1	↓2.0
ENSGALG00000030164	ECH1	Enoyl-CoA hydratase 1, peroxisomal	↓1.2
ENSGALG00000011019	ROR1	Receptor tyrosine kinase-like orphan receptor 1	↓1.2
ENSGALG00000035461	CHORDC1	Cysteine and histidine-rich domain (CHORD) containing 1	↓1.2
ENSGALG00000036044	ALDH3B2	Aldehyde dehydrogenase 3 family member B2	↓1.2
ENSGALG00000032922	GSTM2	Glutathione S-transferase mu 2	↓1.2
ENSGALG00000043088	ROBO4	Roundabout guidance receptor 4	↓1.1
ENSGALG00000011715	HSPA2	Heat shock 70kDa protein 2	↓1.1
ENSGALG00000017122	SGCG	Sarcoglycan, gamma	↓1.1
ENSGALG00000011220	SLC25A29	Solute carrier family 25 member 29	↓1.1

To our surprise, DE analysis identified zero DEGs between LFE and HFE chickens, which exhibited the biggest divergence in abdominal fat content (Table 2). Since FE is a very complex and multifactorial trait, it’s possible that the divergence in the expression profile of abdominal fat between HFE and LFE chickens happened in earlier developmental stages. A newly developed approach also suggests that FE in dairy cattle can be more accurately estimated dynamically, incorporating multiple phenotypic traits throughout various time points [37]. It is likely that future studies could improve the comparison between LFE and HFE chickens through the application of time-series data.

**Fig 4:**
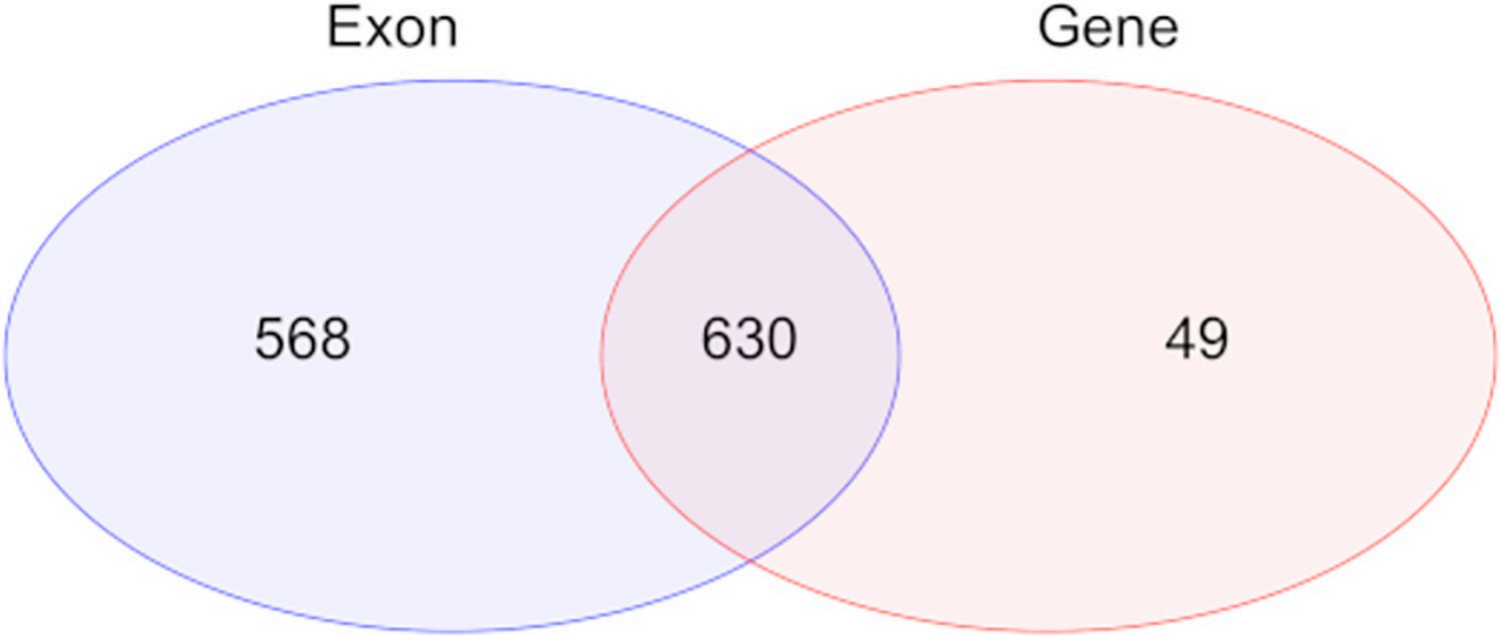
Venn Diagram of DEGs using exon and gene as feature type in categorizing reads by HTseq.

Considering that more than 25% of the 3’ UTR-seq reads were mapped to the intron regions, a separate analysis was conducted by changing the feature type from default “exon” to “gene” in HTSeq, to include both exon- and intron-mapped reads in DE analysis. DE analysis using counts obtained from the *gene* feature type revealed 679 DEGs in total, most of which were in overlap with the results obtained using the feature type “exon” in HTSeq (Fig 4). The additional 49 genes from this analysis (S3 Table) were then added to the whole gene list for functional analysis.

The top 10 ranked pathways identified by ToppFun were shown in Table 4. It seemed that in abdominal adipose tissue, the most prominent difference between HFE and IFE birds occurred in protein anabolism and metabolism. These included pre-mRNA processing (processing of capped intron-containing pre-mRNA), translation initiation (cap-dependent and eukaryotic translation initiation), assembly of ribosome (formation of a pool of free 40S subunits, GTP hydrolysis and joining of the 60S ribosomal subunit) and metabolism of proteins. Although these top ranked pathways were not directly related to lipid accumulation and metabolism, one dissimilarity between the two groups may be the variation in endocrine function of their abdominal fat, as indicated by the pathway L13a-mediated translational silencing of Ceruloplasmin expression, an adipokine recently found over-expressed in adipose tissue of obese subjects [38].

**Table 4:** Top 10 biological pathways identified by ToppFun.

Biological pathways	# Genes in query	# Genes in Pathway	FDR*
Translation	41	165	2.97E-11
Gene Expression	186	1844	4.33E-09
Cap-dependent Translation Initiation	32	127	4.33E-09
Eukaryotic Translation Initiation	32	127	4.33E-09
Metabolism of proteins	168	1631	5.68E-09
GTP hydrolysis and joining of the 60S ribosomal subunit	30	119	1.12E-08
L13a-mediated translational silencing of Ceruloplasmin expression	30	119	1.12E-08
Disease	104	867	1.56E-08
Processing of Capped Intron-Containing Pre-mRNA	45	248	2.26E-08
Formation of a pool of free 40S subunits	27	107	7.55E-08

*FDR: Benjamini–Hochberg false discovery rate calculated by ToppFun.

### Consistent results among Limma, edgeR and Deseq2

**Fig 5:**
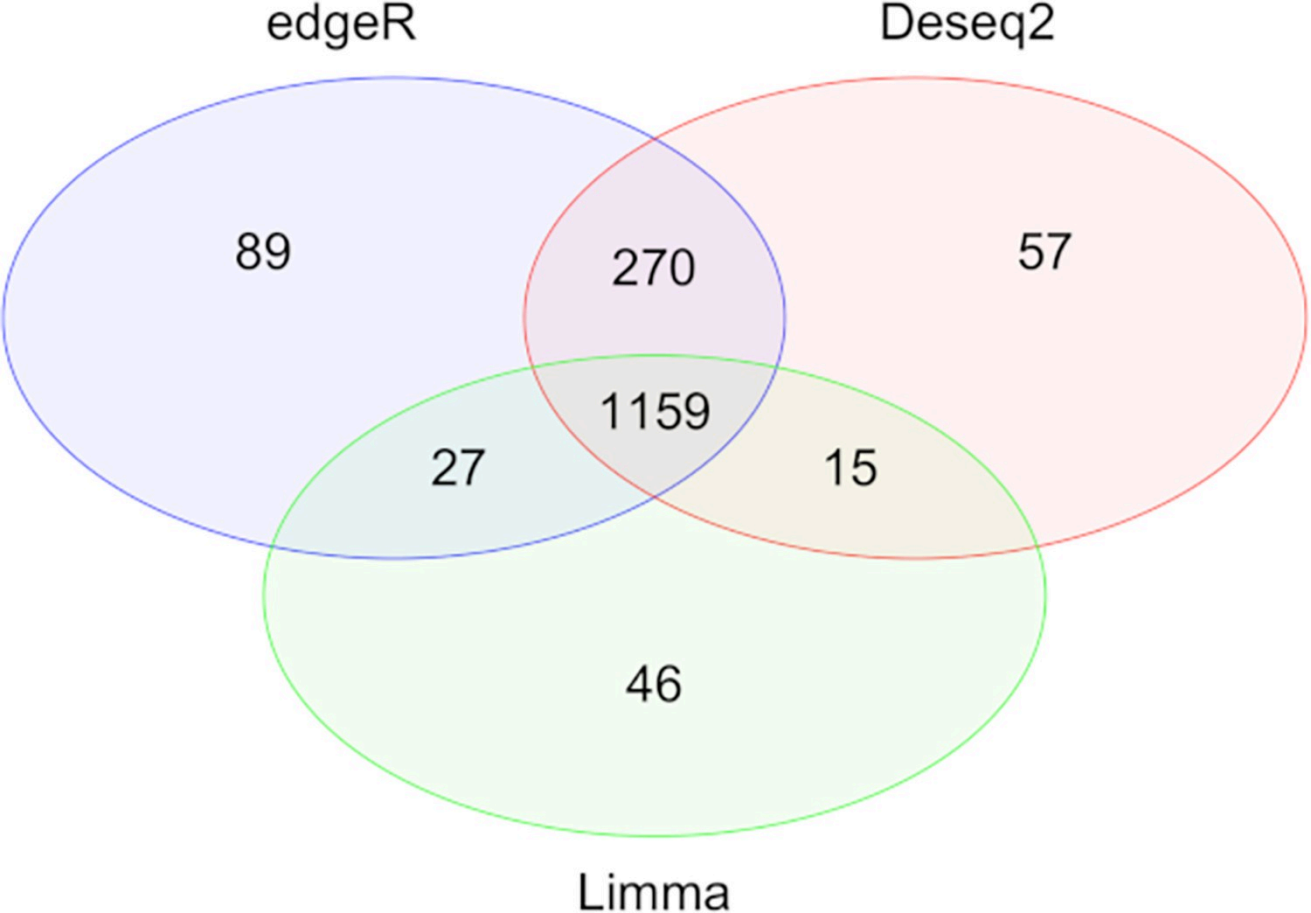
Venn Diagram of DEGs between HFE and IFE chickens obtained from Limma, edgeR and Deseq2.

The results of DE analysis using Limma corroborated the results from edgeR and Deseq2. Combining results obtained using both “exon” and “gene” feature types, there were 1545 and 1501 DEGs determined by edgeR and Deseq2 respectively for the HFE and IFE comparisons. The overlap between the three statistical models was 1159 (Fig 5), manifesting a high level of consistency across these methods. Additionally, for the LFE vs IFE and HFE vs LFE comparisons, edgeR and Deseq2 had comparable performance to Limma as well, with an insignificant number of DEGs detected.

## Discussion

### Intron retention

In general, intron-containing mRNA isoforms are channeled to degradation such as nonsense-mediated decay (NMD) pathway due to the disruption of the main open reading frame (ORF) and following induction of premature termination codons [39]. However, a growing body of research has associated IR with the regulatory role of gene expression, translation, and RNA stability [39]. For example, IR in cytoplasmic mRNA was more frequent across cancer cell lines than normal tissue [40], linking IR to transcriptional diversity in different cancer types and disease states. In addition, Green et al. [41] reported a positive association between decreased IR in nuclear-detained mRNA and enhanced expression of regulators of immunocyte transcription and inflammatory response. IR was even proposed as a biomarker for aging and pre-obese state [42,43].

For a long time, 3’ UTR introns are viewed as marks for nonfunctional transcripts resulting from genomic noise, mutation, and incorrect splicing [44–46]. Nevertheless, more and more studies have noted the possible participation of 3’ UTR intron in regulatory pathways of gene expression. It has been hypothesized that the regulatory function of the 3’ UTR intron was via its interaction with miRNAs, transcriptional and translational factors [47]. Sun et al. determined that IR in 3’ UTR actually increases overall mRNA stability, as NMD was introduced upon splicing of introns in 3’ UTR [48]. A retained intron in 3’ UTR of Calmodulin 3 (Calm3L) mRNA was proved to be a critical binding site to recruit a neuronal double-stranded RNA-binding protein Staufen2 (Stau2), which mediates the dendritic localization of Calm3L mRNA without affecting its stability [49]. Moreover, for activity regulated cytoskeletal associated proteins (Arc), the cis-acting sequences resided primarily in its mRNA 3’ UTR, containing two conserved introns which distinctively modulate stability of Arc mRNA by targeting it for NMD destruction [50]. Upon synaptic signaling, splicing of Arc 3’ UTR introns plays an important role in translational upregulation [50].

Interestingly, a splicing regulator polypyrimidine tract binding protein 1 (PTBP1) was upregulated in IFE chickens by an FC of 1.4. PTBP1 functions in regulating splicing sites of PTBP2, producing PTBP2 transcripts with intron inserted in 3’ UTR [39,44]. During neuron development, PTBP1 was downregulated and the amount of PTBP2 mRNA without intron was thus increased, leading to changes in alternative splicing of proteins involved in neuronal differentiation regulated by PTBP2 [44]. Down-regulation of PTBP1 was also associated with suppressed inflammatory secretome in tumor cells via modulation of intracellular trafficking genes [51], but its effect in adipose tissue remains elusive.

In our study, around 26% of 3’ UTR-seq reads were mapped to introns, indicating that IR events in 3’ UTR were rather frequent in chickens. Up-regulation of alternative splicing regulator PTBP1 in IFE broilers may indicate its involvement in retaining intron in 3’ UTR of mRNAs in avian adipocytes. Therefore, it’s of great interest to further examine the relationship between 3’ UTR IR events and metabolism in chicken adipose tissue in the future.

### Extracellular Matrix (ECM) remodeling

Our DE analysis suggested differences in ECM remodeling in abdominal fat of broilers with different FE levels. ECM dynamics plays an important role in lipid metabolism and protecting adipocytes. First, ECM participates in metabolism and immune responses cell migration, storage of cytokines and binding sites for cellular receptors [52]. Second, adipocytes are embedded in a thick layer of ECM called basal lamina [53], which alleviates mechanical stress to prevent cellular rupture by dispersing the force through this external skeleton [54]. The maintenance of ECM is achieved through its constant turnover and collagen replacement, sustained by a fair investment of metabolic energy [54]. Modification of both amount and makeup of ECM proteins provokes rigidity and adipocyte dysfunction [52,53].

### ECM deposition

Several DEGs identified denoted higher levels of ECM deposition in abdominal adipose tissue of IFE chickens to affect lipid accumulation, including hyaluronidase 2 (HYAL2), hypoxia inducible factor 1 subunit alpha (HIF1α), collagen type III alpha 1 chain (COL3A1), lysyl hydroxylases 2 (PLOD2), and serpin H1 (SERPINH1). One of the top 10 up-regulated genes in HFE group, HYAL2 hydrolyzes hyaluronic acid (HA) of high molecular mass to intermediate HA fragments, which possesses a distinct binding affinity and may be associated with angiogenesis and macrophage infiltration [55]. It has been observed in mice that adipogenesis was accompanied with a net increase in overall HA content [56]. However, a large dose of medium sized HA fibrils was found to hinder lipid accumulation in cultured cells [57]. Consequently, the up-regulation of HYAL2 in the HFE group, with a fold change (FC) close to 3, indicated a restrained lipid accumulation due to higher quantity of intermediate-sized HA molecules.

Additionally, compared with HFE chickens, IFE chickens exhibited a higher expression of HIF1α, PLOD2 and COL3A1 by an average FC of 1.3, further indicating an augmented ECM deposition in their abdominal adipose tissue at 47 days post hatch. Upon expansion, adipose tissue tends to become hypoxic and inflammatory, leading to higher expression of a transcriptional activator HIF1α. In obese mice, transcription level of HIF1α was significantly elevated in adipose tissue [58], as well as other 51 ECM genes stimulated by HIF1α, including COL3A1 and lumican (LUM). This activator also up-regulates PLOD2 under hypoxia, which catalyzes the lysine residue hydroxylation of collagen to hydroxylysine [59]. This hydroxylated residue then binds to lysyl oxidase (LOX) to promote covalent cross-link and collagen glycosylation [59], which stabilize newly formed collagen fibers and increase ECM stiffness [60].

The up-regulation of SERPINH1 in IFE broilers by an FC of 1.6 also indicated the metabolic difference in abdominal fat between IFE and HFE chickens. SERPINH1 possesses collagen-binding properties and participates in production and maturation of ECM collagens [61]. Down-regulation of SERPINH1 in human adipocytes *in vitro* subjected to glucose restriction followed by refeeding suggested its responsiveness to metabolic change caused by weight regain [61].

### Proteoglycans

Several proteoglycans were found differentially expressed in the current study, including endothelial cell-specific molecule 1 (ESM1), agrin (AGRN), platelet derived growth factor subunit B (PDGFB), LUM and syndecans (SDC1, SDC2, SDC4). Besides integrins, proteoglycans are also major ECM receptors in cellular junction and signaling events that regulate metabolic homeostasis and cell fate. It was suggested that ECM receptor interaction may regulate intramuscular fat accumulation in chicken through tissue integrity and signaling transduction [62]. Moreover, composition of ECM and its receptors was found associated with fat depot specific adipogenesis [63].

Out of the three differentially expressed syndecans, of particular interests are SDC1 and SDC4, both up-regulated in IFE chickens with an FC of 2 and 2.2 respectively. SDC1 serves as a lipoprotein uptake receptor and activates PPARγ signaling pathway to initiate adipocyte differentiation [64]. Knock-down of SDC1 in mice can result in depleted lipid deposition in adipose tissue probably via impaired lipid transport and metabolism [64,65]. SDC4 is a typical transmembrane glycoprotein which transmits signals between its core cytoplasmic domain and external chains bound to ECM ligands [66]. The over-expression of SDC4 increases formation of focal adhesion, even without interaction with ECM ligands [66]. Along with several other DEGs including signal transducer and activator of transcription 5A (STAT5A), phosphatase and tensin homolog (PTEN), PDGFB and phosphatidylinositol-4-phosphate 5-kinase type 1 gamma (PIP5K1C), there seemed to be alterations in cell junctions within adipose tissue of HFE and IFE chickens [66–68]. As a result, discrepancies could emerge in cell behavior and tissue integrity which could further affect abdominal fat accumulation [69].

LUM is also up-regulated in the IFE group, involved in collagen repair and innate immune response by facilitating the presentation of bacterial lipopolysaccharide to CD14 [70]. Henegar et.al found evidence of co-expression of LUM and syndecan binding proteins in subcutaneous fat of obese patients [71], as well as metalloproteinases domain 17 (ADAM17) and cytochrome c oxidase assembly homolog (COX17). COX17 is relevant to the functioning of cytochrome c oxidase in electron transfer within the mitochondrial membrane [71]. As expected, ADAM17 and COX17 were down-regulated in the IFE group, with an FC of around 1.6 times lower than the HFE group. Along with 15 differentially expressed mitochondrial ribosomal proteins, out of which 10 were down-regulated in IFE chickens, these DEGs suggested an alteration or even suppression in mitochondrial oxidative activity [72].

### Lipid metabolism

Gene ontology (GO) identified 104 DE genes involved in lipid metabolism, among which 46 were up-regulated and 58 were down-regulated in HFE group. Enriched KEGG pathways included metabolic pathways, peroxisome, FA, and glycerolipid metabolism, among others. Particularly, the suppression of G0/G1 switch gene 2 (G0S2) in HFE chickens could be a critical factor contributing to the variation of the above pathways. Our results complied with a previous study in G0S2 knockout chickens, in which abdominal fat deposition was greatly reduced, along with altered peroxisomal oxidation and triacylglycerol (TAG) synthesis [73].

Peroxisomes are membrane-bound organelles that oxidizes cellular molecules such as FA and thus play an important role in metabolism [74]. 11 DE genes were enriched in peroxisomal pathways, such as peroxisomal biogenesis factor (PEX5, PEX7, PEX16), hydroxysteroid 17-beta dehydrogenase 4 (HSD17B4), sterol carrier protein 2 (SCP2). HSD17B4 catalyzes the oxidation of many lipid intermediates in peroxisomal β-oxidation and SCP2 the subsequent formation of propionyl-CoA [75]. The down-regulation of HSD17B4 and the overexpression of SCP2 in IFE chickens, both by a FC of 1.5, indicate an modified even impaired peroxisomal β-oxidation and possible build-up of toxic lipid intermediates and very long chain FA [76,77]. Moreover, the up-regulation of PEX5, PEX7, PEX16 in IFE chickens suggests an increased biogenesis of peroxisomes [74,78], which could be a remedy attempt for the reduced lipid catabolism. In adipose tissue of low-growth chickens, HSD17B4 was also found down-regulated compared to that of high-growth chickens [8]. These results support the significance of peroxisome in lipid metabolism and HSD17B4 may potentially serve as a biomarker for fat deposition and FE in chicken.

Furthermore, IFE chickens showed signs of disorderly TAG synthesis and lipid metabolism. The rate-limiting enzyme for de novo glycerophospholipid synthesis glycerol-3-phosphate acyltransferase 3 (GPAT3) was down-regulated in IFE chickens by a FC of 1.7. This enzyme catalyzes the first step of TAG synthesis where lysophosphatidic acid (LPA) was synthesized from glycerol-3-phosphate and acyl-CoA [79]. LPA is then converted to phosphatidic acid by 1-acylglycerol-3-phosphate acyltransferases (AGPATs) before the formation of TAG [79]. Unlike GPAT3, AGPAT2 was up-regulated in IFE chickens by a FC of 1.5, marking an imbalance of lipid intermediates in TAG synthesis. On the other hand, differential expression of 3-hydroxyacyl-CoA dehydratase (HACD2, HACD3) and carnitine palmitoyltransferase 2 (CPT2) between HFE and IFE broilers further points to specific differences in lipid metabolism. In addition to impaired peroxisomal FA oxidation, the down-regulation of CPT2 by a FC of 1.6 denotes a decreased obligate step of FA β-oxidation in mitochondria and probably hindered energy expenditure [80]. The up-regulation of the major 3-hydroxyacyl-CoA dehydratase HACD2 by a FC of 1.55, however, implied enhanced FA elongation activity in endoplasmic reticulum [81]. That said, the fat accumulation in IFE chickens may be traced back to disrupted long-chain FA oxidation and production.

## Conclusion

Our results showed a higher variance in sequencing and mapping performance measurements across 3’ UTR-seq samples when compared with RNA-seq, as well as a high correlation between their normalized counts. Moreover, a higher percentage of 3’ UTR-seq reads mapped to introns warrants further research to study intron usage and its regulatory function at the 3’ UTR. Most notably, DE and functional analyses revealed DEGs in the abdominal adipose tissue between HFE and IFE chickens, especially in ECM remodeling, peroxisome, as well as TAG synthesis and lipid metabolism possibly regulated by G0S2. Considering the analogy between chickens and humans in physiological attributes of adipose tissue, the present study could also be applied in the study of adiposity and obesity in humans.

## Supporting information

S1 Fig Correlation plot of production traits. FAT%: Abdominal fat percentage; RFC: Residual feed consumption; FCR: Feed conversion ratio; BMW%: Breast muscle percentage; BW46: Weight at 46 days; FC: Feed consumption.
(TIF)

S1 Table Number of chickens in each FE group from different hatches. (DOCX)

S2 Table Normalized counts of muscle related genes of the 5 samples with possible muscle contamination. (DOCX)

S3 Table Differentially expressed genes between high feed efficiency (HFE) and intermediate feed efficiency (IFE) chickens exclusively found using gene feature type. (DOCX)

S1 Data (DOCX)
