# Supplementary figures and images for: 3’UTR-Seq analysis of chicken abdominal adipose tissue reveals widespread intron retention in 3’UTR and provides insight into molecular basis of feed efficiency
Source: PLoS One. 2022 Jul 1;17(7):e0269534. doi: 10.1371/journal.pone.0269534 (PMC9249230; doi:10.1371/journal.pone.0269534)

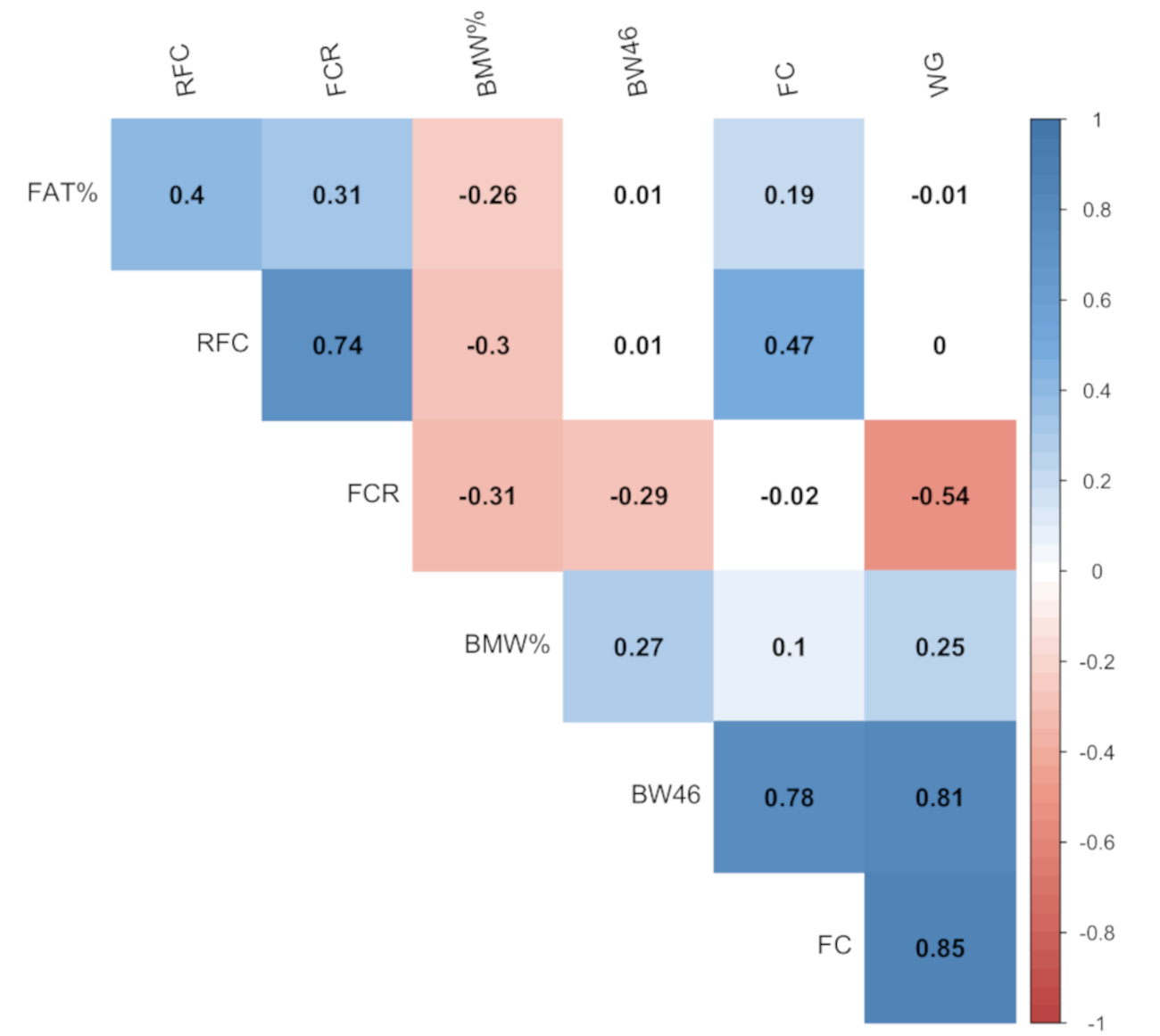

Supplement: S1 Fig — FAT%: Abdominal fat percentage; RFC: Residual feed consumption; FCR: Feed conversion ratio; BMW%: Breast muscle percentage; BW46: Weight at 46 days; FC: Feed consumption. (TIF) [file pone.0269534.s001.tif]
