# Supplementary material for: 3’UTR-Seq analysis of chicken abdominal adipose tissue reveals widespread intron retention in 3’UTR and provides insight into molecular basis of feed efficiency
Source: PLoS One. 2022 Jul 1;17(7):e0269534. doi: 10.1371/journal.pone.0269534 (PMC9249230; doi:10.1371/journal.pone.0269534)
Supplement: S1 Table — (DOCX) [file pone.0269534.s002.docx]

**Table S1. Number of chickens in each FE group from different hatches**

| Hatch | HFE | IFE | LFE |
| --- | --- | --- | --- |
| 1 | 2 | 0 | 2 |
| 2 | 2 | 0 | 2 |
| 3 | 2 | 0 | 2 |
| 4 | 2 | 1 | 3 |
| 5 | 7 | 6 | 6 |
| 6 | 4 | 14 | 5 |
