# Supplementary material for: 3’UTR-Seq analysis of chicken abdominal adipose tissue reveals widespread intron retention in 3’UTR and provides insight into molecular basis of feed efficiency
Source: PLoS One. 2022 Jul 1;17(7):e0269534. doi: 10.1371/journal.pone.0269534 (PMC9249230; doi:10.1371/journal.pone.0269534)
Supplement: S2 Table — (DOCX) [file pone.0269534.s003.docx]

**Table S2. Normalized counts of muscle related genes of the 5 samples with possible muscle contamination**

| Bird ID | MYH1E | ACTA1 | TNNT3 | FE | Hatch |
| --- | --- | --- | --- | --- | --- |
| 47825 | 294.6 | 762.6 | 282.4 | HFE | 5 |
| 38803 | 71.7 | 322.5 | 199.1 | LFE | 4 |
| 47754 | 61.0 | 140.8 | 39.1 | LFE | 6 |
| 47762 | 52.5 | 96.4 | 67.5 | HFE | 6 |
| 47896 | 23.1 | 166.0 | 31.2 | HFE | 5 |
