# Supplementary material for: 3’UTR-Seq analysis of chicken abdominal adipose tissue reveals widespread intron retention in 3’UTR and provides insight into molecular basis of feed efficiency
Source: PLoS One. 2022 Jul 1;17(7):e0269534. doi: 10.1371/journal.pone.0269534 (PMC9249230; doi:10.1371/journal.pone.0269534)
Supplement: S1 Data — (DOCX) [file pone.0269534.s005.docx]

**Supplementary data**

*Fig S1. Correlation plot of production traits.*

*FAT%: abdominal fat percentage; RFC: residual feed consumption; FCR: feed conversion ratio; BMW%: breast muscle percentage; BW46: weight at 46 days; FC: feed consumption.*

**Table S1. Number of chickens in each FE group from different hatches**

| Hatch | HFE | IFE | LFE |
| --- | --- | --- | --- |
| 1 | 2 | 0 | 2 |
| 2 | 2 | 0 | 2 |
| 3 | 2 | 0 | 2 |
| 4 | 2 | 1 | 3 |
| 5 | 7 | 6 | 6 |
| 6 | 4 | 14 | 5 |

**Table S2. Normalized counts of muscle related genes of the 5 samples with muscle contamination**

| Bird ID | MYH1E | ACTA1 | TNNT3 | FE | Hatch |
| --- | --- | --- | --- | --- | --- |
| 47825 | 294.6 | 762.6 | 282.4 | HFE | 5 |
| 38803 | 71.7 | 322.5 | 199.1 | LFE | 4 |
| 47754 | 61.0 | 140.8 | 39.1 | LFE | 6 |
| 47762 | 52.5 | 96.4 | 67.5 | HFE | 6 |
| 47896 | 23.1 | 166.0 | 31.2 | HFE | 5 |

**Table S3. Differentially expressed genes between high feed efficiency (HFE) and IFE (intermediate) chickens exclusively found using gene feature type**

| Ensembl ID | Gene Symbol | Gene Full Name | Log2FC |
| --- | --- | --- | --- |
| ENSGALG00000048482 | - | - | ↑3.3 |
| ENSGALG00000047727 | **-** | **-** | ↑2.9 |
| ENSGALG00000055126 | - | - | ↑2.2 |
| ENSGALG00000046990 | **-** | **-** | ↑2 |
| ENSGALG00000050517 | - | - | ↑1.8 |
| ENSGALG00000053949 | **-** | **-** | ↑1.8 |
| ENSGALG00000048812 | - | - | ↑1.7 |
| ENSGALG00000053008 | **-** | **-** | ↑1.2 |
| ENSGALG00000032419 | - | - | ↑1 |
| ENSGALG00000052503 | **-** | **-** | ↑0.8 |
| ENSGALG00000050734 | - | - | ↑0.7 |
| ENSGALG00000049825 | **-** | **-** | ↑0.6 |
| ENSGALG00000052558 | - | - | ↑0.5 |
| ENSGALG00000051765 | **-** | **-** | ↓0.8 |
| ENSGALG00000040489 | - | - | ↓1.5 |
| ENSGALG00000046164 | APBB1 | Amyloid Beta Precursor Protein Binding Family B Member 1 | ↓1.3 |
| ENSGALG00000016602 | ARHGAP6 | Rho GTPase Activating Protein 6 | ↑0.6 |
| ENSGALG00000017326 | ARHGEF17 | Rho Guanine Nucleotide Exchange Factor 17 | ↑0.7 |
| ENSGALG00000038469 | CDC25A | Cell Division Cycle 25A | ↑0.9 |
| ENSGALG00000008969 | EPCAM | Epithelial Cell Adhesion Molecule | ↑0.8 |
| ENSGALG00000036391 | GFPT1 | Glutamine--Fructose-6-Phosphate Transaminase 1 | ↑0.9 |
| ENSGALG00000033888 | IDH3B | Isocitrate Dehydrogenase (NAD(+)) 3 Non-Catalytic Subunit Beta | ↑0.6 |
| ENSGALG00000013372 | IL7R | Interleukin 7 Receptor | ↓0.9 |
| ENSGALG00000015391 | KCMF1 | Potassium Channel Modulatory Factor 1 | ↑0.4 |
| ENSGALG00000007641 | LDB1 | LIM Domain Binding 1 | ↑1.1 |
| ENSGALG00000051398 | LOC420860 | - | ↓0.5 |
| ENSGALG00000037787 | MEF2D | Myocyte Enhancer Factor 2D | ↑0.4 |
| ENSGALG00000009013 | MKKS | MKKS Centrosomal Shuttling Protein | ↓0.5 |
| ENSGALG00000009974 | MMAA | Metabolism Of Cobalamin Associated A | ↑0.8 |
| ENSGALG00000023670 | NAALADL2 | N-Acetylated Alpha-Linked Acidic Dipeptidase Like 2 | ↑2.3 |
| ENSGALG00000013155 | PCBP2 | Poly(RC) Binding Protein 2 | ↑0.6 |
| ENSGALG00000011192 | PHF10 | PHD Finger Protein 10 | ↑0.6 |
| ENSGALG00000007970 | PIAS1 | Protein Inhibitor Of Activated STAT 1 | ↑0.4 |
| ENSGALG00000044574 | PLPP5 | Phospholipid Phosphatase 5 | ↑0.8 |
| ENSGALG00000034572 | POC1A | POC1 Centriolar Protein A | ↑0.8 |
| ENSGALG00000013383 | PPIE | Peptidylprolyl Isomerase E | ↑0.6 |
| ENSGALG00000003634 | PTEN | Phosphatase And Tensin Homolog | ↓0.5 |
| ENSGALG00000031440 | PTPRU | Protein Tyrosine Phosphatase Receptor Type U | ↑1.5 |
| ENSGALG00000009202 | RBM45 | RNA Binding Motif Protein 45 | ↑0.5 |
| ENSGALG00000040184 | RIMKLB | Ribosomal Modification Protein RimK Like Family Member B | ↑0.4 |
| ENSGALG00000004140 | SH3BP4 | SH3 Domain Binding Protein 4 | ↑0.9 |
| ENSGALG00000013995 | TAAR5 | Trace Amine Associated Receptor 5 | ↑2.2 |
| ENSGALG00000042739 | TACC1 | Transforming Acidic Coiled-Coil Containing Protein 1 | ↑0.5 |
| ENSGALG00000040017 | THRA | Thyroid Hormone Receptor Alpha | ↑0.5 |
| ENSGALG00000051415 | TM2D1 | TM2 Domain Containing 1 | ↓0.7 |
| ENSGALG00000037629 | TRANK1 | Tetratricopeptide Repeat And Ankyrin Repeat Containing 1 | ↑0.6 |
| ENSGALG00000011660 | WTAP | WT1 Associated Protein | ↑0.5 |
| ENSGALG00000028998 | XPA | XPA, DNA Damage Recognition And Repair Factor | ↑0.4 |
| ENSGALG00000030487 | ZXDC | ZXD Family Zinc Finger C | ↑0.8 |

↑: Up-regulated in HFE chickens; ↓: down-regulated in HFE chickens
